# Roadblocks in Chagas disease care in endemic and nonendemic countries: Argentina, Colombia, Spain, and the United States. The NET-Heart project

**DOI:** 10.1371/journal.pntd.0009954

**Published:** 2021-12-30

**Authors:** Andrés F. Miranda-Arboleda, Ezequiel José Zaidel, Rachel Marcus, María Jesús Pinazo, Luis Eduardo Echeverría, Clara Saldarriaga, Álvaro Sosa Liprandi, Adrián Baranchuk, on behalf of the Neglected Tropical Diseases and other Infectious Diseases affecting the Heart (NET-Heart) project

**Affiliations:** 1 Cardiology Department, Pablo Tobón Uribe Hospital, Medellín, Colombia; 2 Division of Cardiology, Kingston Health Science Centre, Queen’s University, Kingston, Ontario, Canada; 3 Cardiology Department, Sanatorio Güemes, and School of Medicine, University of Buenos Aires, Buenos Aires, Argentina; 4 LASOCHA, Washington, DC, United States of America; 5 Medstar Union Memorial Hospital, Baltimore, Maryland, United States of America; 6 ISGlobal, Hospital Clinic, University of Barcelona, Barcelona, Spain; 7 Cardiology Department, Cardiovascular Foundation of Colombia, Floridablanca, Colombia; 8 Cardiology Service, Clínica CardioVID, Universidad de Antioquia, Medellín, Colombia; Instituto de Investigaciones Biotecnológicas, ARGENTINA

## Abstract

**Background:**

Chagas disease (CD) is endemic in Latin America; however, its spread to nontropical areas has raised global interest in this condition. Barriers in access to early diagnosis and treatment of both acute and chronic infection and their complications have led to an increasing disease burden outside of Latin America. Our goal was to identify those barriers and to perform an additional analysis of them based on the Inter American Society of Cardiology (SIAC) and the World Heart Federation (WHF) Chagas Roadmap, at a country level in Argentina, Colombia, Spain, and the United States, which serve as representatives of endemic and nonendemic countries.

**Methodology and principal findings:**

This is a nonsystematic review of articles published in indexed journals from 1955 to 2021 and of gray literature (local health organizations guidelines, local policies, blogs, and media). We classified barriers to access care as (i) existing difficulties limiting healthcare access; (ii) lack of awareness about CD and its complications; (iii) poor transmission control (vectorial and nonvectorial); (iv) scarce availability of antitrypanosomal drugs; and (v) cultural beliefs and stigma. Region-specific barriers may limit the implementation of roadmaps and require the application of tailored strategies to improve access to appropriate care.

**Conclusions:**

Multiple barriers negatively impact the prognosis of CD. Identification of these roadblocks both nationally and globally is important to guide development of appropriate policies and public health programs to reduce the global burden of this disease.

## Author summary

Chagas disease (CD) has been described as an epidemic in Latin America, but its geographical influence is global. One of the biggest challenges in providing care for patients with CD is to improve access to early diagnosis and treatment in order to avoid chronic cardiovascular and gastrointestinal complications. However, different roadblocks interfere with the optimal care of these patients, which facilitates disease progression. While some barriers to care are global in scope, there are additionally national and even local obstacles for patients with CD. Appropriate delineation of these barriers will allow for the development of targeted interventions to improve the outlook for CD patients in both endemic and nonendemic countries alike.

## Introduction

**Fig 1:**
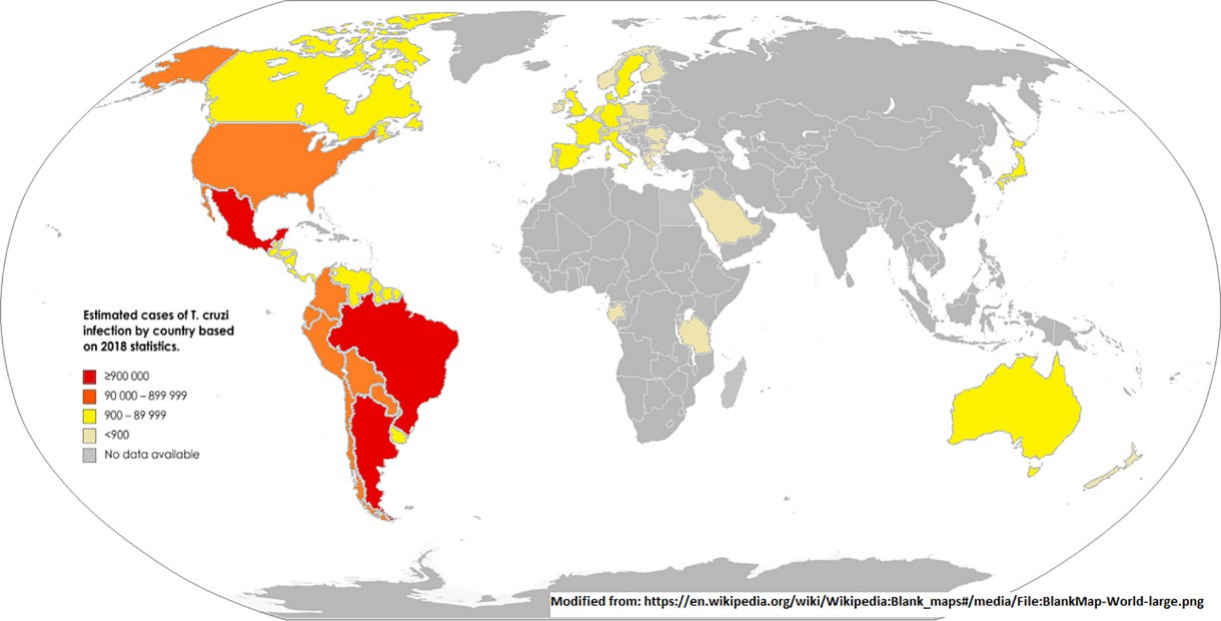
Global epidemiology of Chagas disease. Data from World Health Organization Control of Tropical Neglected Disease. Modified from: https://en.wikipedia.org/wiki/Wikipedia:Blank_maps#/media/File:BlankMap-World-large.png.

Chagas disease (CD) is both preventable and treatable; however, the number of infected individuals worldwide remains approximately 6 to 7 million. Numerous barriers to care are responsible for this impressive figure (**Fig 1**) [1]. In a recent document, the Inter American Society of Cardiology (SIAC) and the World Heart Federation (WHF) delineated the obstacles to providing effective care for this neglected tropical disease (NTD) [2]. That document functioned as a frame of reference and was not intended to address country-specific barriers.

Argentina and Colombia are 2 upper-middle-income countries that have had resources and programs for the eradication of CD for decades. Spain is a nonendemic country, but large-scale immigration from endemic countries in Latin America has introduced CD and transmission by nonvectorial routes. Finally, there has been a growing understanding that CD poses an important problem in the United States (US), chiefly as a disease in immigrants, but also with an as-of-yet incompletely understood burden of domestically acquired disease [3].

The SIAC’s NET-Heart project is a program that evaluates current evidence about NTDs and other infectious diseases with cardiac manifestations [4]. Gathering experts from the SIAC-WHF roadmap and NET-Heart project, we aimed to analyze and classify current barriers for optimal care for CD in endemic and nonendemic countries.

## Methods

We performed a nonsystematic review of literature published in indexed journals from 1955 to 2021 and a selection of gray literature including abstracts, local guidelines issued by health entities, blogs, media, and local policies. Lists of references were used to complete the search. Experts representing SIAC NET-Heart project [4] and the WHF Roadmap [2], from 2 endemic countries (Argentina and Colombia) as well as 2 nonendemic countries (Spain and the US) performed a detailed analysis of national barriers based on data identified, knowledge of the disease, and local context. Barriers were classified, and potential solutions were proposed.

## Results

### Situation analysis of CD in specific countries

**Fig 2:**
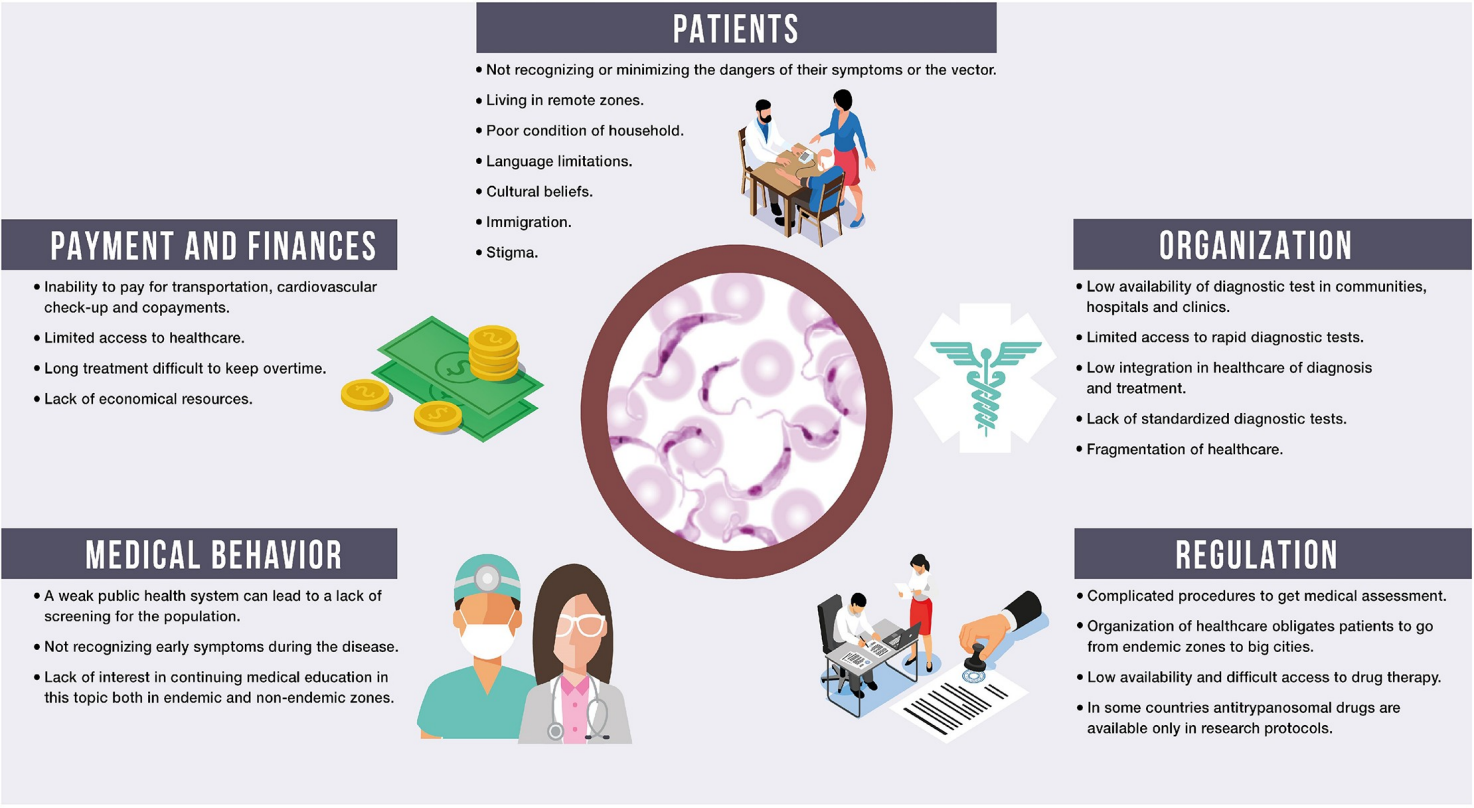
Barriers and roadblocks limiting Chagas disease care.

The SIAC-WHF roadmap classified roadblocks in 4 main clinical areas: prevention, diagnosis, treatment of CD, and diagnosis and treatment of disease complications. An analysis of these barriers by country is tabulated below (**Fig 2 and Table 1**).

**Table 1:** Specific roadblocks by country to access optimal care of CD integrated with SIAC-WHF roadmap.

Barriers and potential solutions	Argentina	Colombia	Spain	USA
**Prevention**	Insufficient screening, no prioritization even in endemic provinces. Lack of implementation of home spraying programs.	Insufficient serologic testing to screen asymptomatic patients.	Insufficient awareness and lack of national guidelines for prenatal testing. Ignorance or denial of the disease by patients.	Limited vector surveillance. No oral route detected. Incomplete testing in organ donors.
**Diagnosis**	Lack of availability of diagnostic tests in rural areas. Analysis in noncertified laboratories, heterogeneous cutoff points for tests. PCR is not available.	Any serologic test for CD or its complications is available in endemic areas. Lack of classification and availability of rapid tests. Patients’ copayments. Highly specialized care is remote from rural communities.	Serologic testing for *T*. *cruzi* is carried out in expert centers only. Despite having health insurance, many undocumented immigrants do not access the health system.	Diverse immigrant population with testing well validated only for *T*. *cruzi* II. Difficult to access confirmatory testing. Cost of testing is prohibitive. Undocumented individuals have very limited access to care.
**Treatment**	Intermittent availability of Nifurtimox. Difficulties in accessing Benznidazole.	Delays in importation of trypanocidal drugs, treatment reserves are sometimes scarce. Delivery of drugs to patients requires a laborious and bureaucratic process.	Imported drugs, bureaucratic procedures that make immediate access to drugs difficult.	Recent approval of Benznidazole and Nifurtimox. Lack of familiarity with medications among providers.
**Treatment of complications**	Disconnection between primary care and rural physicians with specialists in treating complications. Insufficient access to medication, implantable heart devices, and heart transplantation.	Complications are usually treated and followed in tertiary care hospitals, far away from endemic areas. Lack of awareness of complications symptomatology and clinical findings.	Lack of knowledge of progression of cardiac and digestive complications by specialists, as well as the efficacy of treatments.	Lack of awareness among cardiologists of CD and its treatment. Undocumented individuals have varying but generally poor access to care.

CD, Chagas disease; PCR, polymerase chain reaction.

References used to create the table are as follows: Echeverria LE, Marcus R, Novick G, Sosa-Estani S, Ralston K, Zaidel EJ, et al. WHF IASC Roadmap on Chagas Disease. Glob Heart. 2020;15(1):26 [2]; Forsyth C, Meymandi S, Moss I, Cone J, Cohen R, Batista C. Proposed multidimensional framework for understanding Chagas disease healthcare barriers in the United States. PLoS Negl Trop Dis; 2019;13(9):e0007447 [5]; Manne-Goehler J, Reich MR, Wirtz VJ. Access to care for Chagas disease in the United States: a health systems analysis. Am J Trop Med Hyg. 2015;93(1):108–13 [6].

### Current situation of CD in Argentina

In Argentina, a marked decrease in vectorial transmission has been achieved since the early 1990s via the international collaborative project INCOSUR [7]. Despite this, Argentina is the country where most CD patients live worldwide, with approximately 1 case of CD for every 27 Argentinians [2]. Health personnel are chiefly located in cities where there are not vectors (“vinchucas”), and have little awareness of CD, so it remains undiagnosed for decades [2].

Rural-to-urban migration and the reduction of vectorial transmission have made vertical transmission the main route of infection in the country. In local estimates, the proportion of pregnant women with positive Chagas serology was significantly reduced over time, but the proportion of infected neonates remained stable [8].

Diagnostic lab tests have variable accuracy, even in blood banks, where CD screening is mandated and must then communicated to a seropositive donor [9]. However, there is no oversight for this screening process, and positive cases may not receive either confirmation or access to treatment.

Currently, in Argentina, both adult dose and pediatric dose benznidazole is produced domestically and is widely available; it is also exported internationally. Nifurtimox, however, is imported, and its supply is interrupted intermittently. A pediatric dosage is under development in the country [10].

Recently, the Ministry of Health published updated guidelines for the diagnosis and management of CD [11]. Unfortunately, these recommendations have not been fully disseminated or implemented, mainly due to fragmented provincial legislation and a concomitant shift in focus away from Chagas due to the Coronavirus Disease 2019 (COVID-19) pandemic.

Gastrointestinal complications are infrequent in Argentina, but simultaneously underrecognized. Cardiovascular complications are widely recognized in both endemic areas and in urban centers. Nevertheless, because of the lack of a strong Chagas-specific evidence base, uncertainty about the efficacy of pharmacologic and device therapy for Chagas heart disease compared to other heart diseases means a lack of a “standard of care.”

One of the main determinants of the persistence of CD is that it is neglected by most policy makers. The budget for CD was less than 3% of what is allocated to political publicity campaigns and governmental events, and of what is allocated, less than 50% is implemented. CD requires long-term health policies that are difficult to implement in a rapidly changing political scenario [12].

### Current situation of CD in Colombia

In Colombia, it is estimated that 2% of the population has CD, with regional rates as high as 7%. The most affected age group are working-aged adults. Universal screening for CD in blood donors has been mandated by law since 1995, and seroprevalence based on blood bank data is approximately 0.5% [13].

Zones of higher prevalence are characterized by domestic and peridomestic cycles of *T*. *cruzi*, higher rates of natural infection in triatomines, and nonhuman mammal species that serve as natural reservoirs [13].

As an endemic country, vectorial transmission continues to be a problem, with rates up to 23.1 cases/1,000 habitants in high-endemicity locales. Appreciation of the importance of oral transmission has increased in urban and rural areas; outbreaks around the country have been attributed to ingestion of beverages or food contaminated with *T*. *cruzi*. Oral infection causes a more severe acute presentation, with rapid evolution to complications such as myocarditis, and is associated with increased mortality [14].

Since the inception of the national CD control program in 1996, Colombia has continuously made efforts to control CD: Program goals are to clarify the national epidemiological status; identify the distribution of vectors, index of domiciliary infection, rates of infection in school children, and conditions of housing; and prioritize efforts to control the disease. In 2007, the Ministry of Social Protection updated the National Guidelines for the diagnosis and management of patients with CD [15].

Eradication of CD has been an area of interest for the Colombian government; the country is part of the Andean Initiative to Control Vectorial and Transfusional Transmission of CD and Medical Care of CD (IPA). Due to this initiative, by 2018, Colombia achieved interruption of vectorial transmission in more than 23 municipalities and 4 departments [16,17].

Colombia is currently implementing the Strategy for the Integrated Management of Health Promotion, Prevention, and Control of Vector-Transmitted Diseases for 2012–2021, which aims for a reduction of 40% of social, clinical, and economic consequences of vector-borne diseases including CD [18].

### Current situation of CD in Spain

According to the Spanish National Institute of Statistics, the number of foreign nationals living in Spain exceeded 5 million in 2020 [19,20]. Over the last 20 years, there has been an exponential increase in the number of migrants from Latin America, and many diagnoses of CD have been reported in an area considered to be nonendemic, which challenges the concept of endemicity itself.

Spain is currently the European country with the greatest number of estimated cases of CD in absolute numbers (between 48,000 and 86,000 people) [21] and in percentage (between 2.7% and 4.9% of the Latin American population) [22].

Prior analyses of barriers to care in CD have highlighted a lack of knowledge among health professionals, but there are few studies evaluating awareness of CD among healthcare professionals. Based on the study by Ramos-Rincón and colleagues, the level of knowledge of CD among healthcare professionals and students in a specific territory in Spain was adequate but could be improved [23].

One of the main challenges in Spain is the significant underdiagnosis of the disease. Despite evidence suggesting the cost-effectiveness of screening [24], it is voluntary and is not widespread in Spain. National regulation regarding screening in pregnancy to prevent congenital disease has been established, and in autonomous regions like Catalonia and Valencia, control measures for *T*. *cruzi* infection in pregnant women at risk and control programs of newborns have already been approved [25]. Spain’s ministry of health requires screening for *T*. *cruzi* infection before organ donation from immigrants as well as children born to mothers from endemic countries [26].

Clinical guidelines and consensus documents have been published to provide protocols for the care of chronic Chagas cardiovascular and digestive disease. [27–30] Recommendations about the management of CD in primary care [31] and in the immunosuppressed patient have also been published [32,33].

The 2 antiparasitic drugs used currently to treat *T*. *cruzi* infection (benznidazole and nifurtimox) are available in Spain. However, because they are imported, there is limited access due to (1) requirement that a hospital-based pharmacy disburse the medication; and (2) bureaucratic procedures that delay the immediate start of treatment [34].

Several studies focus on the social dimension of CD and describe the complex psychosocial barriers [35]. CD diagnosis has caused social and labor discrimination in endemic countries, and at-risk individuals postpone diagnosis/treatment due to lack of economic resources, fear, and even the lack of interest of some toward a disease that manifests mainly in the long term [35].

In nonendemic countries, migrants at risk for CD face similar concerns [36,37]. People residing legally in Spain have access to universal healthcare through the National Health System, theoretically facilitating treatment, but barriers such as work schedules, legal issues, language barriers, unfamiliarity with rights, social exclusion, and direct and indirect discrimination hinder their care [38].

During the last decade, as these barriers have been identified, patient advocacy organizations have organized to demand easier access to care for individuals with CD [39,40].

### Current situation of CD in the US

Despite its ability to provide state-of-the-art medical treatment, the US remains a difficult place in which to receive appropriate care for CD. There are approximately 300,000 patients with CD in the US [41]. There are numerous barriers to care that can be grouped into 4 categories:

Lack of awareness of CD is profound in the US, both in the at-risk community and in the medical community from whom it receives care. While immigrants from regions in countries in which there are a very high prevalence of CD may recognize the disease name and pathology, immigrants from other countries, notably Mexico and El Salvador, frequently have not heard of the disease [42], which complicates their ability to advocate for screening and treatment. Medical schools in the US do not recognize CD as a diagnosis that is likely to be encountered domestically, and poor knowledge of the disease promotes resistance to screening efforts and recognition of the disease when it is manifest. Recently, American Heart Association (AHA) launched a scientific statement regarding all aspects of the management of cardiac complications of CD [43], but for busy clinicians who do not think that they will see patients with this disease, this resource is likely underutilized.

Individuals with CD in the US are chiefly foreign-born immigrants; while both reduviid bugs and *T*. *cruzi* are found within the lower half of the US, transmission appears to be very rare. Individuals with CD are frequently non-English speakers, indigent, uninsured, and undocumented. The medical communities that care for them are also frequently low-resource settings, in which the ability to address a complicated disease is difficult and potentially cost-prohibitive [6].

The recent approval of both benznidazole and nifurtimox have eased access to therapeutics, but the complexity of this disease and the lack of robust data to support either cure or reduction in cardiac events for most infected individuals is a barrier to the adoption of widespread screening. Testing for CD in the diverse immigrant population in the US, infected with both *T*. *cruzi* I and *T*. *cruzi* II sp., highlights the lack of quality diagnostics that have been proven to perform accurately in patients infected with either parasite strain [44].

Finally, there are structural problems that complicate the appropriate delivery of care in the US. Successful models of screening and treatment programs exist, but in states that have beneficent healthcare programs for immigrants, even those without documentation. These cannot be easily extrapolated to states where this is not the case [5]. Furthermore, the state public health labs frequently play a role in testing, particularly as the Centers for Disease Control and Prevention (CDC) is currently the only institution that can provide confirmatory testing. Each state, and often each county, may have a different policy about how to deal with these labs. Few states mandate reporting of CD. Medical care in the US is also guideline-driven, and without US-based professional societies endorsing screening, it is very difficult to effectively promote CD screening programs [45].

## Discussion

We have found diverse barriers that make CD difficult to manage, many of which are found in both endemic and nonendemic environments. The main barriers identified through this review were organization of and access to the healthcare system, medical behavior, lack of awareness, and sociocultural aspects. Our analysis, nevertheless, showed that while similar, roadblocks manifest differently in different countries and require regional strategies for better control of CD. Regardless, increased awareness of the disease and its manifestations, coupled with appropriate guidelines about screening and treatment, could synergistically overcome those barriers globally.

With regard to financing, CD received 0.67% of the total financing of all NTD during 10 years of assessment, so it earns its description as “the most neglected of the neglected” NTD [46]. CD causes a loss of 752,000 days of work every year due to premature death, with an average cost of 1.2 billion dollars per year in southern countries of Latin America [47].

Since the arrival of the SARS-CoV-2 pandemic in late 2019, many vector eradication, screening, and diagnostic programs, as well as trypanocidal treatments, have been downscaled. A recently published document covers the possible interactions between COVID-19 and CD, as well as the potential negative impact on all aspects of CD caused directly or indirectly by the pandemic. If CD was always considered a neglected disease, in the context of a pandemic, it is even more so [48].

In this paper, our goal was to search for globally relevant barriers, then align corrective measures for them with the specific needs of endemic and nonendemic countries using 2 different countries as models for every case. We then integrated our findings with strategies proposed by the SIAC-WHF roadmap pointing to reduce the global impact of CD, along with the WHO objectives for 2030 to improve access to trypanocidal treatment, interrupt vectorial transmission and vertical transmission, and eliminate transmission related to blood transfusions and tissue transplantation in targeted countries [1].

To improve the care of CD patients, different policies must be implemented in endemic and nonendemic countries. Improved access to screening and diagnosis in populations at risk, specifically serological testing in remote areas, implementation of screening in at-risk pregnant women, blood banks, and tissue donors will improve the possibility of appropriate intervention to mitigate disease. Better integration between community and rural hospitals and the tertiary care institutions required to care for patients with significant cardiovascular or gastrointestinal complications will improve outcomes. Ongoing educational programming will help health professionals involved in the care of CD need to be able to recognize its risk factors, clinical presentation, complications, screening indications, and treatment. Economic barriers are an important hindrance to the optimal care of CD. Reducing copayments, strengthening local programs to avoid the need of patients traveling to main cities, and guaranteeing access to “essential” medication will ameliorate the economic barriers to care.

Finally, a collaborative effort by policy makers, healthcare providers, and patients should strive to eradicate sociocultural barriers. Educational programs in endemic regions focusing on prevention, recognition of the vector and its link with CD, improved/upgraded housing construction, and integration of indigenous communities are all factors that must be taken into account. In nonendemic countries, ameliorating language barriers will reduce stigma generated by this condition. The WHO’s work to establish World Chagas Disease Day (April 14th) is a positive step toward raising awareness of the issues described in this review, but the current barriers to care for those affected by CD have prompted the Chagas coalition [49], the WHF, and SIAC to make a strong call for immediate action.

### Limitations

Access to care for individuals with CD is evolving, and some of the identified barriers may change in a short period of time. Some of the local policies and strategies to address CD care are only published by local governmental health entities of each country. This issue may impact the ability to find the sources of information.

This manuscript did not include any authors from Brazil, which is one of the most affected countries in South America.

## Conclusions

Despite its discovery and comprehensive description more than 100 years ago, multiple barriers continue to adversely impact the prognosis and global burden of CD. Identification of these roadblocks globally and regionally is necessary to improve the implementation of policies and strategies for both prevention and treatment of this potentially catastrophic disease.

**Key Learning Points**

- Chagas disease (CD) is endemic in Latin America, but it has become a problem of global magnitude.
- The clinical outcomes of CD are influenced by a complex interplay of political, social, cultural, economic, and environmental factors. At each of these levels, there are barriers that limit healthcare for patients with this condition.
- The main barriers are payment and financing, organization and regulation of the health system, medical behavior, and sociocultural aspects.
- Recognizing national and global barriers will allow the development of adjustable strategies to mitigate the impact of this condition.
- Optimal care and control of CD is multimodality and multidisciplinary and involves all the actors of the system to overcome each of the barriers detected.

**Top Five Papers**

1. Echavarría NG, Echeverría LE, Stewart M, Gallego C, Saldarriaga C. Chagas Disease: Chronic Chagas Cardiomyopathy. Curr Probl Cardiol. 2021 Mar;46(3):100507. doi: 10.1016/j.cpcardiol.2019.100507
2. Alonso-Padilla J, Cortés-Serra N, Pinazo MJ, Bottazzi ME, Abril M, Barreira F, et al. Strategies to enhance access to diagnosis and treatment for Chagas disease patients in Latin America. Expert Rev Anti Infect Ther. 2019 Mar;17(3):145–157.
3. Echeverría LE, Marcus R, Novick G, Sosa-Estani S, Ralston K, Zaidel EJ, et al. WHF IASC Roadmap on Chagas Disease. Glob Heart. 2020 Mar 30;15(1):26. doi: 10.5334/gh.484
4. Burgos LM, Farina J, Liendro MC, Saldarriaga C, Liprandi AS, Wyss F, et al. Neglected Tropical Diseases and other Infectious Diseases affecting the Heart (NET-Heart project). Neglected Tropical Diseases and Other Infectious Diseases Affecting the Heart. The NET-Heart Project: Rationale and Design. Glob Heart. 2020 Sep 1;15(1):60.
5. Mills RM. Chagas Disease: Epidemiology and Barriers to Treatment. Am J Med. 2020 Nov;133(11):1262–1265. doi: 10.1016/j.amjmed.2020.05.022
